# Virologic outcome among patients receiving antiretroviral therapy at five hospitals in Haiti

**DOI:** 10.1371/journal.pone.0192077

**Published:** 2018-01-30

**Authors:** Frantz Jean Louis, Josiane Buteau, Kesner François, Erin Hulland, Jean Wysler Domerçant, Chunfu Yang, Jacques Boncy, Robert Burris, Valerie Pelletier, Nicholas Wagar, Varough Deyde, David W. Lowrance, Macarthur Charles

**Affiliations:** 1 Centers for Disease Control and Prevention, Port-au-Prince, Haiti; 2 Laboratoire National de Santé Publique, Ministère de la Santé Publique et de la Population, Port-au-Prince, Haiti; 3 Programme National de Lutte contre le SIDA, Ministère de la Santé Publique et de la Population, Port-au-Prince, Haiti; 4 Centers for Disease Control and Prevention, Atlanta, Georgia, United States of America; National and Kapodistrian University of Athens, GREECE

## Abstract

**Introduction:**

Viral load (VL) assessment is the preferred method for diagnosing and confirming virologic failure for patients on antiretroviral therapy (ART). We conducted a retrospective cross-sectional study to evaluate the virologic suppression rate among patients on ART for ≥6 months in five hospitals around Port-au-Prince, Haiti.

**Methods:**

Plasma VL was measured and patients with VL <1,000 copies/mL were defined as virologically suppressed. A second VL test was performed within at least six months of the first test. Factors associated with virologic suppression were analyzed using logistic regression models accounting for site-level clustering using complex survey procedures.

**Results:**

Data were analyzed for 2,313 patients on ART for six months or longer between July 2013 and February 2015. Among them, 1,563 (67.6%) achieved virologic suppression at the first VL test. A second VL test was performed within at least six months for 718 (31.0%) of the patients. Of the 459 patients with an initial HIV-1 RNA <1,000 copies/mL who had a second VL performed, 394 (85.8%) maintained virologic suppression. Virologic suppression was negatively associated with male gender (adjusted odds ratio [aOR]: 0.80, 95% CI: 0.74–0.0.86), 23 to 35 months on ART (aOR:0.72[0.54–0.96]), baseline CD4 counts of 201–500 cells/mm^3^ and 200 cells/mm^3^ or lower (aORs: 0.77 [0.62–0.95] and 0.80 [0.66–0.98], respectively), poor adherence (aOR: 0.69 [0.59–0.81]), and TB co-infection (aOR: 0.73 [0.55–0.97]).

**Conclusions:**

This study showed that over two-thirds of the patients in this evaluation achieved virologic suppression after ≥ six months on ART and the majority of them remained suppressed. These results reinforce the importance of expanding access to HIV-1 viral load testing in Haiti for monitoring ART outcomes.

## Introduction

Routine HIV-1 viral load monitoring is essential for ensuring optimal treatment outcomes of patients on antiretroviral therapy (ART) and preventing the emergence and transmission of HIV drug resistance (HIVDR). While there has been a major expansion of ART in most resource-limited settings, access to routine virologic monitoring remains limited [1]. In Haiti, in the last 10 years with major support from the United States President's Emergency Plan for AIDS Relief (PEPFAR) and the Global Fund for AIDS, Tuberculosis, and Malaria, over 85,000 HIV-infected patients, representing 60% of the 141,000 estimated persons living with HIV/AIDS (PLHIV) in Haiti are enrolled in antiretroviral therapy (ART) [2]. Despite the Haiti national HIV care and treatment guidelines recommending viral load (VL) monitoring for patients on ART, VL testing had not been routinely performed before 2016 due to limited laboratory capacity, lack of resources, and the technical challenges encountered during testing [3]. Patients were monitored clinically and immunologically (CD4 T-cell count), according to the World Health Organization (WHO) criteria [4]. The pitfalls of this approach are that clinical and CD4 T-cell count criteria are poor predictors of antiretroviral treatment failure [5–8]. Patients may appear clinically stable with a robust CD4 T-cell count while they are virologically failing their ART (virologic failure is defined as having HIV-1 RNA ≥1000 copies/mL at least six months after initiation of ART); conversely, patients may show signs of clinical deterioration with a decrease in CD4 T-cell count, while their VL remains suppressed (Virologic suppression is defined as having a HIV-1 RNA <1000 copies/mL at least six months after initiation of ART) [6, 9, 10]. The lack of sensitivity and specificity of this approach has been shown to lead to delayed recognition of virologic failure and unnecessary switching to second-line regimens. This could result in the emergence and accumulation of drug resistance (DR) mutations or patients being prescribed more toxic and expensive regimens, further limiting drug options for the patients and increasing overall ART program cost [5].

Routine VL monitoring for HIV patients on ART is a standard practice in resource-rich countries [11–13]. Many studies have consistently shown the benefits of VL monitoring [10, 14, 15]. More importantly, at the individual patient level, sustained virologic suppression can prevent the emergence of DR mutations and decrease the risk of clinical therapeutic failure [16, 17]. Patients who achieve early virologic suppression remain on first-line antiretroviral regimens longer, have a lower risk of developing opportunistic infections, and face lower mortality rate [5, 15]. From the HIV prevention and public health significance, sustained virologic suppression at the community level can substantially decrease HIV transmission and reduce new HIV infections [18]. Currently, there are limited data on the proportion of patients who are failing first-line ART and the rate of switching to second line regimens in Haiti. The fact that 95% of ART-patients are still receiving first-line regimens may indicate that some patients are receiving ineffective antiretroviral drugs [19]. With efforts to scale up treatment and attain the ambitious UNAIDS “90-90-90” target by 2020, it is paramount to develop and implement strategies that ensure patients have access to virologic monitoring [20]. In addition, the proportion of patients achieving virologic suppression after ART initiation is an important measure of programmatic success. Here we present virologic outcomes during a multi-center evaluation of patients receiving ART for at least six months at five major hospitals in the Port-au-Prince metropolitan area.

## Materials and methods

### Study design and ART sites

This is a retrospective cross-sectional study design. The facilities included in this evaluation were: Hôpital de l’Université d’État d’Haïti, Hôpital Isaïe Jeanty-Léon Audain, Hôpital Universitaire La Paix, Grace Children’s Hospital, and Hôpital Saint Damien-Nos Petits Frères et Soeurs. These are five major hospitals in the Port-au-Prince metropolitan area that provide care to approximately 10% of all active patients on ART in the country.

### Patient population and ART

HIV-positive patients received care according to the Haiti national HIV care and treatment guidelines [3]. The first-line ARV regimens in Haiti include two nucleoside reverse transcriptase inhibitors (NRTIs) and one non-nucleoside reverse transcriptase inhibitor (NNRTI): tenofovir/zidovudine, lamivudine, and efavirenz/nevirapine. The Haiti guidelines recommend VL monitoring for patients receiving ART for six months or more. However, testing has not been available due to limited capacity. In 2012, with PEPFAR support, in anticipation of VL access expansion across the country, the National HIV Control Program undertook a pilot VL testing at five major hospitals in the Port-au-Prince metropolitan area. In this pilot study, all HIV-positive patients receiving ART for at least six months at these five hospitals were eligible to be included. Patients with a HIV-1 RNA ≥1,000 copies/mL received intensive adherence counselling for three months after the first VL test. A second follow-up VL test was performed within at least six months of the first test. All the VL test results were captured into iSanté, a web-based electronic medical record system [21].

### Plasma HIV-1 viral load measurement

Plasma VL was measured using the Generic HIV Viral Load Assay (Biocentric, Bandol, France) following the validated procedure in place at the National Public Health Laboratory in Haiti. In this assay, the limits of detection are 300-10^7^ copies/mL when 0.2 mL plasma volumes are used for testing [22].

### Data collection

Individual patient demographic and clinic data were abstracted at each of the five hospitals from the web-based electronic medical record system, iSanté, for all patients enrolled from July 2013 to February 2015. The demographic data collected included gender and age. Clinical data included WHO clinical stages at the time of the first blood collection for VL test, tuberculosis co-infection, antiretroviral regimen usage, duration of ART in years, and adherence history. Adherence was assessed using a standardized questionnaire with information on patients’ knowledge of the disease, pill counts, ART side effects, stigma, and respect of clinic appointments [23]. An adherence score of 90% or less was considered as poor adherence while >90% was considered good [24, 25]. Laboratory data included baseline CD4 T-cell counts at ART initiation, the first VL for all patients on ART for at least six months, and second VL at least six months after the first one. All duplicate entries were removed; patients with missing ART information, erroneous VL data, or had conflicting information were also removed from analyses.

### Statistical analysis

Descriptive statistics were generated for demographic and clinical parameters. To account for large amounts of missing data for key variables, multiple imputations were performed using fully conditional specification discriminant functions and logistic regression models for categorical variables and predictive mean matching regression for continuous variables. Missing data were assumed missing at random (MAR), and, due to moderately high levels of missing data, 10 imputations were performed. The imputation models included all demographic and clinical covariables, hospitals, and the outcome variable of VL, for which we had a complete dataset for all individuals in the study. Unadjusted and adjusted logistic regression models using multiple imputed data and complex survey procedures accounting for site-level clustering were used to describe the association between clinical and demographic factors with the odds of having a HIV-1 RNA level <1,000 copies/mL (viral load suppression). ART interruption was not documented and was not taken into account in the data analysis. Both SAS version 9.3 (SAS Institute, Cary, NC) and SPSS Version 20.0 (SPSS, v20.0 Chicago: SPSS Inc.) were used for analyses.

### Ethical review

The Haiti National Bioethics Committee and the Centers for Disease Control and Prevention (CDC) approved the protocol for this project and determined it to be a public health program evaluation. Therefore, Institutional Review Board (IRB) review and informed consent were not required, as the activity did not constitute human subjects research. Data were fully anonymized before they were extracted for analysis.

## Results

### Demographic and clinical characteristics

Table 1 displays both original and multiple imputed demographic and clinical characteristics of the patients at the time of their first VL testing, except for CD4 counts for which we collected baseline CD4 data at ART initiation. After imputation, the median age was 39.0 years (interquartile range [IQR] 29.0–47.0). Of the 2,313 participants, 61.9% were women. The median CD4 T-cell count was 329.0 cells/mm^3^ (IQR 183.0–593.0). TB co-infection was observed in 20.7% of patients, whereas 51.8% of the patients were at WHO stage II and 57.1% had an adherence score of 90% or more, indicating good adherence.

**Table 1:** Demographic, clinical, virologic, and immunological characteristics of study participants at the time of the first viral load testing (n = 2,313).

		Original Data			Following Multiple Imputation
Characteristics		N or Median	Total N	% or IQR	% or Median (IQR)
**Gender**	M	868	2,278	38.1	38.1
	F	1,410	2,278	61.9	61.9
	Observations missing data	35	2,313	1.5	
**Age (Years)**	Median age	39.0	2,292	29.0–47.0	39.0 (29.0–47.0)
	0–12	245	2,292	10.7	11.0
	13–21	116	2,292	5.1	5.1
	22–40	926	2,292	40.4	40.3
	41–50	590	2,292	25.7	25.7
	>50	415	2,292	18.1	18.0
	Observations missing data	21	2,313	0.9	
**Time on ART (Months)**	Median Months	29.0	2,313	16.0–52.0	-
	<12 Months	365	2,313	15.8	-
	12–23 Months	543	2,313	23.5	-
	24–35 Months	445	2,313	19.2	-
	36–47 Months	268	2,313	11.6	-
	48–59 Months	298	2,313	12.9	-
	60 + Months	394	2,313	17.0	-
**Baseline CD4 T-cell count (cells/mm^3^)**	Median count	329.0	1,839	183.0–593.0	329.0 (183.0–585.0)
	≤200	517	1,839	28.1	28.4
	201–500	736	1,839	40.0	40.4
	>500	586	1,839	31.9	31.3
	Observations missing data	474	2,313	20.5	
**WHO Stage**	I	136	943	14.4	14.8
	II	529	943	56.1	51.8
	III	237	943	25.1	29.4
	IV	41	943	4.4	3.9
	Observations missing data	1,370	2,313	59.2	
**ART Regimen**	TDF-3TC-EFV	576	2,280	25.3	25.1
	TDF-3TC-NVP	116	2,280	5.1	5.1
	ZDV-3TC-EFV	816	2,280	35.8	35.9
	ZDV-3TC-NVP	571	2,280	25.0	25.1
	PI-based	58	2,280	2.5	2.5
	Other	143	2,280	6.3	6.3
	Observations missing data	33	2,313	1.4	
**Viral load**^a^ **(Copies/mL)**	Overall median	300	2,313	300.0–5327.7	-
	Median among those with detectable VL^b^	19,822.8	891	2,029.2–125,967.3	-
	Median among VL <1000 and detectable VL^b^	574.2	141	413.1–729.5	-
	Median among VL ≥1000	34,667.9	750	6,204.9–168,192.1	-
**Adherence**	Adherence Median	100.0	1,225	90.0–100.0	100.0 (90.0–100.0)
	Poor	527	1,225	43.0	42.9
	Good	698	1,225	57.0	57.1
	Observations missing data	1,088	2,313	47.0	
**TB co-infection**	No	1,465	1,880	77.9	79.3
	Yes	415	1,880	22.1	20.7
	Observations missing data	433	2,313	18.7	
**Hospitals**^a^	Hospital 1	927	2,313	40.0	-
	Hospital 2	296	2,313	12.8	-
	Hospital 3	580	2,313	25.1	-
	Hospital 4	87	2,313	3.8	-
	Hospital 5	423	2,313	18.3	-

^**a**^ Viral load, time on ART, and hospital site were not missing any data in the original dataset and were not multiply imputed.

^b^ Detectable viral load is defined as viral loads greater than 300 copies/mL. This excluded 1,422 observations.

### Antiretroviral drug regimens

The majority (91.2%) of the participants were treated with a regimen containing two NRTIs and one NNRTI in the following combinations: Tenofovir (TDF) or Zidovudine (ZDV)/lamivudine (3TC)/Efavirenz (EFV), (61.0%), and TDF or ZDV/3TC/Nevirapine (NVP), (30.2%). The remaining participants were either on protease inhibitors (PI)-based regimens (2.5%) or other regimens (6.3%). The median duration on ART was 29.0 months (IQR 16.0–47.0) and 84.2% of the patients had been on ART for longer than one year.

### Virologic outcomes

#### Initial viral load determination

**Fig 1:**
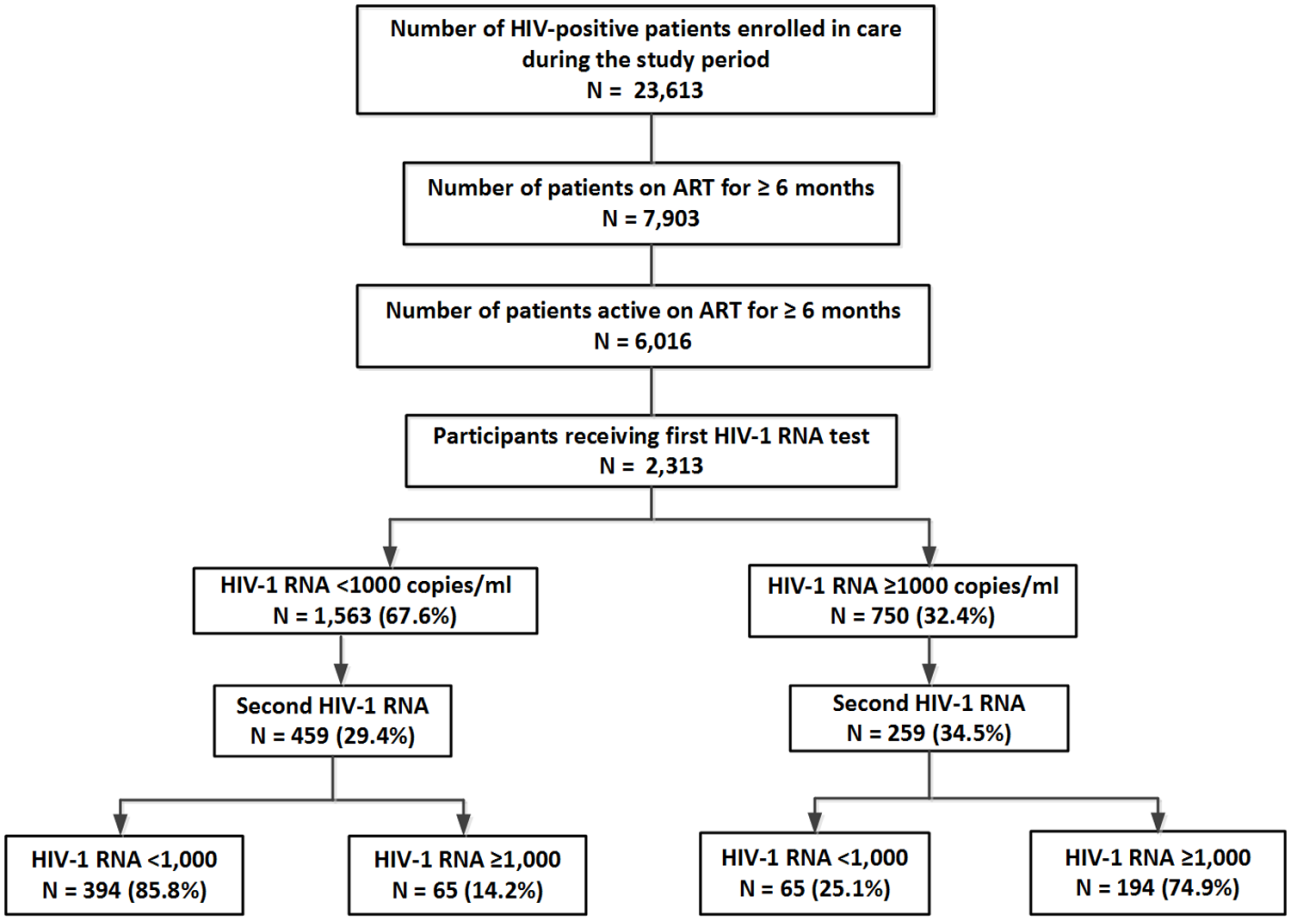
Flowchart describing the number of patients with first and second VL test and percent of virologic suppression among them.

Plasma VL tests were performed for all the 2,313 patients at their first VL measurement time point (Fig 1 and Table 1), which was at minimum six months after ART initiation. The median time from ART initiation to the first VL test was 29 months (IQR: 16–52 months). Over two-thirds (67.6%, N = 1,563) of the patients had plasma HIV-1 RNA <1000 copies/mL. Among those with HIV-1 RNA <1000 copies/mL, 91.0% (N = 1,422) had a VL of <300 copies/mL, which was the lower limit of detection for the VL assay used; 9.0% (N = 141) of the patients had a VL between 300 and 1,000 copies/mL. Furthermore, 32.4% (N = 750) of the patients had a HIV-1 RNA ≥1000 copies/mL. The median VL for patients with HIV-1 RNA ≥1000 copies/mL was 34,667.9 copies/mL (IQR: 6,204.9–168,192.1) while that for patients with HIV-1 RNA between >300 and <1000 copies/mL was 574.2 (IQR 413.1–729.5) (Table 1).

#### Follow-up viral load determination

A second VL test was performed for 718 (31.0%) of the 2,313 participants. Of the 459 patients with an initial HIV-1 RNA <1,000 copies/mL who had a second VL performed, 85.8% (394) maintained virologic suppression (Fig 1). Of the 259 patients with initial HIV-1 RNA ≥1,000 copies/mL who had a second VL test 25.1% (N = 65) achieved virologic suppression following intensive adherence counseling while the remaining patients (N = 194, 74.9%) had two consecutive HIV-1 RNA ≥1,000 copies/mL even after intensive adherence counseling (Fig 1). The median time between the first and second VL tests was 8.0 months (IQR: 7.0–11.0 months); at the time of the second VL test, patients had been on ART for a median of 39.0 months (IQR: 27.0–60.0 months).

#### Factors associated with virologic outcomes

To investigate whether virologic suppression was associated with demographic and clinical characteristics or antiretroviral regimens used and treatment facilities, we performed univariable and multivariable logistic regression analyses using the dataset after multiple imputation corrections for missing data (Table 2). In the univariable analyses, adults aged >50 years were significantly more likely to achieve virologic suppression than those aged 22–40 years (OR: 2.21, 95% CI: 1.80–2.70), as were those patients with a WHO clinical stage II when compared to those with stage I (OR: 1.49, 95% CI: 1.06–2.09). Compared to patients on ZDV-3TC-EFV, those on ZDV-3TC-NVP and PI-based regimens were significantly less likely to achieve virologic suppression (OR: 0.73, 95% CI: 0.63–0.85 and OR: 0.31, 95% CI: 0.18–0.55, respectively). Patients with poor adherence (OR: 0.63 [0.54–0.74]), males (OR: 0.90 [0.83–0.97]), and those having been on ART for 24–35 months (OR: 0.67 [0.55–0.81]) were all significantly less likely to achieve virologic suppression.

**Table 2:** Factors associated with viral suppression using univariable and multivariable logistic regression models.

			Univariable analysis		Multivariable analysis	
Covariables	Level	% Virologic suppression^a^	OR^b^	95% CI	aOR^c^	95% CI ^d^
**Gender**	M	66.0	**0.90**	**0.83–0.97**	**0.80**	**0.74–0.86**
	F	68.5	1.00	-	1.00	-
**Age (Years)**	0–12	62.0	0.95	0.58–1.58	0.91	0.54–1.52
	13–21	50.0	0.58	0.23–1.42	0.52	0.22–1.26
	22–40	63.5	1.00	-	1.00	-
	41–50	71.7	1.45	0.95–2.22	**1.45**	**1.01–2.08**
	>50	79.2	**2.21**	**1.80–2.70**	**2.07**	**1.70–2.51**
**Time on ART (Years)**	<12 Months	70.1	1.00	-	1.00	-
	12–23 Months	65.6	0.81	0.64–1.03	0.83	0.59–1.15
	24–35 Months	61.1	**0.67**	**0.55–0.81**	**0.72**	**0.54–0.96**
	36–47 Months	64.9	0.79	0.61–1.01	0.79	0.57–1.09
	48–59 Months	69.1	0.95	0.77–1.18	0.95	0.60–1.51
	60 + Months	75.9	1.34	0.99–1.81	1.36	0.86–2.15
**Baseline CD4 T-cell count**	≤200	66.8	0.92	0.72–1.18	**0.77**	**0.62–0.95**
	201–500	67.2	0.94	0.79–1.12	**0.80**	**0.66–0.98**
	>500	68.8	1.00	-	1.00	-
**WHO Stage**	IV	68.0	1.34	0.67–2.66	1.4	0.66–2.95
	III	64.7	1.14	0.75–1.73	1.11	0.70–1.77
	II	71.0	**1.49**	**1.06–2.09**	**1.47**	**1.03–2.10**
	I	61.1	1.00	-	1.00	-
**ART Regimen**	ZDV-3TC-EFV	71.5	1.00	-	1.00	-
	TDF-3TC-EFV	68.8	0.88	0.64–1.20	0.86	0.57–1.28
	TDF-3TC-NVP	66.5	0.80	0.39–1.64	0.92	0.42–2.00
	ZDV-3TC-NVP	64.7	**0.73**	**0.63–0.85**	**0.69**	**0.57–0.83**
	PI-based	43.8	**0.31**	**0.18–0.55**	**0.35**	**0.20–0.59**
	Other	62.7	0.67	0.35–1.28	0.80	0.45–1.44
**Adherence**	Poor	62.0	**0.63**	**0.54–0.74**	**0.69**	**0.59–0.81**
	Good	71.8	1.00	-	1.00	-
**TB co-infection**	Yes	62.7	0.77	0.58–1.02	**0.73**	**0.55–0.97**
	No	68.8	1.00	-	1.00	-

Those in bold type indicate significance of < 0.05.

^**a**^ Percent suppressed was calculated within each level of each covariable using multiply imputed data.

^**b**^ OR: Odds Ratio

^**c**^ aOR: Adjusted Odds Ratio

^**d**^ CI: Confidence interval

The multivariable analysis revealed that older adults aged 41–50 years and ≥50 years had 1.45 times (95% CI: 1.02–2.08) and 2.07 times (95% CI: 1.70–2.51) the odds of virologic suppression when compared to those aged 22–40 years. Male gender was associated with a 20% decrease in odds of virologic suppression compared to females (aOR: 0.80, 95% CI: 0.74–0.86). Those with TB co-infection had 27% decreased odds of virologic suppression (aOR: 0.73 [0.55–0.97]) and those with poor adherence had 31% decreased odds of virologic suppression (aOR: 0.69 [0.59–0.81]). Those with decreased CD4 counts had lower odds of virologic suppression when compared to those with CD4 counts of 500 cells/mm^3^ or greater (aOR: 0.77 [0.62–0.95] for 200 cells/ mm^3^ or fewer; aOR: 0.80 [0.66–0.98] for 201–500 cells/mm^3^). Receiving ZDV-3TC-NVP regimens (aOR: 0.70, 95% CI 0.54–0.89) or PI-based regimens (aOR: 0.32, 95% CI 0.18–0.56) were significantly associated with a plasma HIV-1 RNA ≥1000 copies/mL when compared to ZDV-3TC-EFV; TDF-3TC-EFV regimens, TDF-3TC-NVP regimens and other regimens did not differ significantly from ZDV-3TC-EFV regimens. Having been on ART for 24–35 months was associated with a 28% decrease in odds of virologic suppression when compared to those on ART for fewer than 12 months (aOR: 0.72 [0.54–0.96]). Finally, WHO stage II presentation was associated with 1.47 times the odds of virologic suppression when compared to those presenting with WHO Stage I (95% CI: 1.03–2.10); there was no significant difference in odds of virologic suppression for those patients with WHO stages III or IV when compared to stage I (Table 2).

## Discussion

Implementation of routine VL monitoring for patients on ART has been the main focus in most resource-limited countries since the publication of the WHO 2013 consolidated ARV guidelines [1]. The Ministry of Health of Haiti adopted the WHO recommendations in 2013. However, until 2016, implementation of routine VL monitoring in Haiti’s national ART program has been hampered by the lack of high throughput VL testing platforms and limited human resources. This is the first multi-center evaluation of ART outcomes for patients on ART. The results of this evaluation will guide the ongoing implementation and scale up of routine VL monitoring at the national level. The challenges and factors associated with virologic suppression identified in this study will inform the expansion of routine VL testing efforts aimed at achieving the third of UNAIDS’ 90-90-90 goal leading to epidemic control.

In the current evaluation, we found that about 68% of the participants on ART for ≥6 months achieved virologic suppression. These results are similar to those reported in other resource-limited countries [26–28]. A previous study at the GHESKIO Clinic in Haiti also showed that 38.3% (57/149) of the patients on ART for ≥12 months had virologic failure [16]. The results of the current study underscore the need for routine VL monitoring for patients on ART and to improve ART program performance in resource-limited settings including Haiti.

Studies have indicated that many demographic and clinical parameters may impact treatment outcomes for ART patients [27, 29–32]. The current evaluation also revealed that adolescents and young adults have greater risk of virologic failure, consistent with other reports suggesting that this age group faces multiple social, psychological and adherence challenges increasing their vulnerability to treatment failure [27–29]. TB co-infection has been reported as a predictor of virologic failure which is consistent with our findings [30–32]. This finding supports the strong recommendation of the Haiti National HIV Treatment guidelines to actively screen PLHIV for TB [3]. Our findings also highlight the importance of enhanced adherence counseling to sustain virologic suppression [33]. Indeed, 86% of patients with two consecutive VL maintained virologic suppression and of those with an initial HIV-1 RNA ≥ 1000 copies/mL, 25% achieved virologic suppression after intensive adherence counseling. The Haiti national HIV care and treatment guidelines recommend a second VL test for all patients with an initial HIV-1 RNA ≥ 1000 copies/mL within three to six months. However, only 259 out of the 750 patients (34.5%) with initial virologic failure benefited from a second VL measurement. These results underlie the need to reinforce training for healthcare providers on the importance of VL in monitoring patients on ART. Therapeutic education should also be provided for patients as part of their care package, so they can better understand their results and help generate the demand for VL testing.

WHO now recommends treating all PLHIV regardless of CD4 T cell count and the Ministry of Health of Haiti has recently adopted the ‘test and start’ strategy [34]. In light of the ‘test and start’ implementation in Haiti and the findings of the current study that patients with poor adherence, TB-co-infections, children and adolescents were less likely to achieve virologic suppression (Table 2), implementation of routine VL monitoring will ensure early identification of factors that might prevent the attainment of the third 90 target and lead to the development of strategies to overcome those obstacles. With more than 95% of PLHIV in Haiti still receiving first-line regimens, and in light of the virologic failure rate observed in this evaluation, reinforcing viral load monitoring is essential for early detection of treatment failure and for proper timing of the transition to second-line regimens.

The findings of this evaluation should be interpreted with caution in light of the limitations. First, the cross-sectional design and the lack of geographic representativeness of the study warrant caution in the interpretation and generalization of the findings. Second, healthcare providers might have prioritized patients with suspected treatment failure since this was the first time that VL testing was offered to patients at these health facilities, which may have led to a selection bias towards a higher rate of virologic failure. Third, while missing at random was assumed, these data may be missing in a pattern correlated to virologic suppression and may have introduced bias into the study. Finally, the WHO guidelines recommend a second VL test be performed after 3 months of intensive adherence counseling, in this study however, the second VL test was performed within at least six months after the initial VL test, which makes it difficult to accurately evaluate the impact of adherence on virologic suppression.

## Conclusions

This is the first multicenter evaluation on virologic outcome of patients receiving ART in Haiti. The results provide important information about the rate of virologic suppression, showing that a high proportion of patients maintain virologic suppression and underscoring the importance of intensive adherence counseling. Our findings will be important to inform the current scaling up of routine viral load monitoring in Haiti and the revision of the National HIV treatment guidelines.
